# Collateral sensitivity: An evolutionary trade‐off between antibiotic resistance mechanisms, attractive for dealing with drug‐resistance crisis

**DOI:** 10.1002/hsr2.1418

**Published:** 2023-07-11

**Authors:** Mina Yekani, Robab Azargun, Simin Sharifi, Edris Nabizadeh, Javid Sadri Nahand, Navideh Karimi Ansari, Mohammad Yousef Memar, Jozsef' Soki

**Affiliations:** ^1^ Department of Microbiology, Faculty of Medicine Kashan University of Medical Sciences Kashan Iran; ^2^ Infectious and Tropical Diseases Research Center Tabriz University of Medical Sciences Tabriz Iran; ^3^ Student Research Committee Kashan University of Medical Sciences Kashan Iran; ^4^ Department of Microbiology, Faculty of Medicine Maragheh University of Medical Science Maragheh Iran; ^5^ Dental and Periodontal Research Center Tabriz University of Medical Sciences Tabriz Iran; ^6^ Department of Microbiology, Faculty of Medicine Tabriz University of Medical Sciences Tabriz Iran; ^7^ Institute of Medical Microbiology, Albert Szent‐Györgyi Faculty of Medicine University of Szeged Szeged Hungary

**Keywords:** antimicrobial agents, collateral sensitivity, combination therapy

## Abstract

**Background:**

The discovery and development of antimicrobial drugs were one of the most significant advances in medicine, but the evolution of microbial resistance limited the efficiency of these drugs.

**Aim:**

This paper reviews the collateral sensitivity in bacteria and its potential and limitation as a new target for treating infections.

**Results and Discussion:**

Knowledge mechanisms of resistance to antimicrobial agents are useful to trace a practical approach to treat and control of resistant pathogens. The effect of a resistance mechanism to certain antibiotics on the susceptibility or resistance to other drugs is a key point that may be helpful for applying a strategy to control resistance challenges. In an evolutionary trade‐off known as collateral sensitivity, the resistance mechanism to a certain drug may be mediated by the hypersensitivity to other drugs. Collateral sensitivity has been described for different drugs in various bacteria, but the molecular mechanisms affecting susceptibility are not well demonstrated. Collateral sensitivity could be studied to detect its potential in the battle against resistance crisis as well as in the treatment of pathogens adapting to antibiotics. Collateral sensitivity‐based antimicrobial therapy may have the potential to limit the emergence of antibiotic resistance.

## 1 INTRODUCTION

The evolution of resistance to antimicrobial agents in bacterial pathogens reflects an increasing global health challenge and emphasizes the need to introduce new treatment procedures for infectious diseases.^1^, ^2^, ^3^ Antimicrobial resistance often arises by several mechanism including decrease the intracellular levels of antibiotics, drug target site modification or bypass, and enzymatically inactivation.^4^, ^5^ Resistance is a result of mutation that provides a competitive superiority for mutated sub‐populations in response to the selective pressure following the use of antimicrobial agents.^6^, ^7^ The development and spread of antibiotic resistant bacteria are a universal threat to humans and animals. The drug‐resistance crisis is generally not preventable but can be controlled, and it must be limited by practical procedure.^8^, ^9^ Current drug discovery pipelines of new‐in‐class antibiotic agents are insufficient to offset the emergence of new antimicrobial resistance.^10^, ^11^ Introducing new antibiotics is not a common approach, and decreased sensitivity has expanded to various antimicrobial agents, regardless of their molecular structures and microbial targets. Innovative strategies to reduce the rate that at antimicrobial resistance develops are thus critically needed.^12^, ^13^ Knowledge the mechanism of resistance to antibiotics is essential for detecting and discovering novel antimicrobial drugs as well as the control of resistant pathogens in health care centers.^14^ The impact of a mechanism of resistance to an agent on the evolution of susceptibility to other antibiotics is an important question that may be applied to reach strategy against resistance challenge.^15^ In a phenomenon called collateral sensitivity, the increasing resistance to one antibiotic is associated with increased susceptibility to second antimicrobial agents. Collateral sensitivity has been reported in various clinically important pathogens and between a wide spectrum of antimicrobial agents. However, the molecular mechanisms which increase the susceptibility of bacteria to secondary antimicrobial agents have not been well described in more cases.^16^ A programmed increase in antimicrobial susceptibility can improve the efficiency of antimicrobial therapy. Thus, the question emerges whether the collateral sensitivity is used to overcome the antibiotic resistance crisis and eradicate pathogens adapting to different antimicrobial agents. Theoretically, infections caused by resistant bacteria may be treated by a combination of antibiotics with collateral sensitivity or by cycling an antibiotic to another that bacteria display collateral sensitivity to it. This review focuses on introducing the collateral sensitivity and its potential as a new potential strategy for treating of infections.

## 2 METHODOLOGY

In the present literature review, data on various aspects of collateral sensitivity in bacteria and its potential and limitation as a new target for treating infections were detected in databases of PubMed, Scopus and the Google Scholar. The internet searches were done to find published manuscripts with the keyword's antibiotics resistance, antimicrobial agents, collateral sensitivity, combination therapy. All English language articles were found and read independently by two individuals. Overview of strategy in literature search for data including inclusion/exclusion criteria and results was provided in Supporting Information: Figure S‐1.

## 3 RESULTS AND DISCUSSION

### 3.1 The mechanisms of resistance to antimicrobial agents

Microbial drug resistance can be due to intrinsic, acquired, or adaptive mechanisms. Bacteria may be intrinsically non‐susceptible to given drugs and also can be resistant due to acquiring genes encoding resistance mechanisms by horizontal gene transfer (HGT) pathways or mutations in the chromosome.^5^, ^17^ In intrinsic resistance to antibiotics, a certain group of bacteria has inheritable molecular or functional features which increase the resistance to the antimicrobial activity of a particular drug.^18^ The very known example of intrinsic resistance to antibiotics is the deficiency in the uptake of an agent due to the specific structural or physiological features of bacteria, such as resistance of Gram‐negative bacteria to vancomycin and resistance of anaerobic bacteria to aminoglycosides.^19^, ^20^ The production of a Metallo‐β‐lactamase that mediates the resistance of *Stenotrophomonas maltophilia* to carbapenems is other example of an intrinsic resistance. Acquired resistance is results of the HGT and mutation in a previously sensitive bacterium. HGT can occur through three main mechanisms of DNA transfer pathways included transformation, transduction and conjugation. Adaptive resistance is mediated by some particular environmental stimuli such as stress, growth state, pH, ions concentrations, starvation, subinhibitory levels of antimicrobial agents. Adaptive resistance allows microorganisms to respond more quickly to antimicrobial stress. It usually is transient, and regresses to the primary state after removing the stimuli signal.^21^

**Figure 1:**
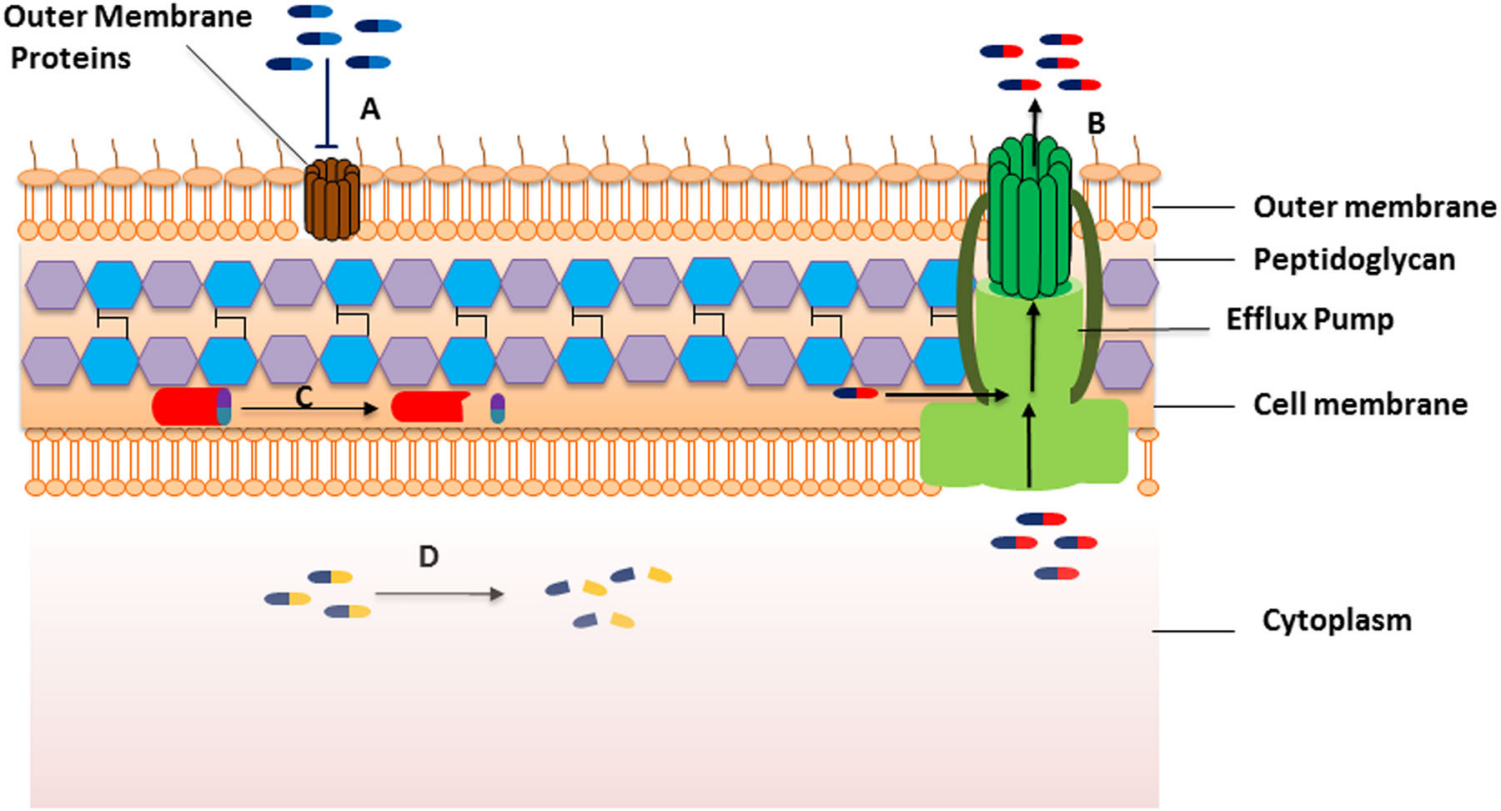
Mechanisms of antibiotic resistance: Mutation in the outer membrane proteins (A) and overexpression of efflux pump (B) decrease the antibiotic concentration in bacteria. Modifying of the antibiotic target (C) through mutation or post‐translational modification can confer resistance. Inactivation of antibiotics by hydrolysis or transfer of a chemical group (D) by bacterial enzymes causes antibiotic resistance.

Resistance of bacteria to antibiotics is mediated by different molecular mechanisms that fall into three primary ones (Figure 1): first, mechanisms that decrease the intracellular levels of the drugs, such as decreased permeability or pump out toxic agents. For example, mutation in the outer membrane proteins (OMPs) and overexpression of MexAB efflux pumps decrease the carbapenems concentration in *Pseudomonas aeruginosa*.^22^ Second, resistance mediated by modifying the drug target through mutation or post‐translational modification such as resistance to methicillin in *Staphylococcus aureus* that is conferred by PBP‐2a with significantly decreased affinity to β‐lactams.^23^ Third, mechanisms of inactivate the antimicrobial agent by enzymatic modification, rendering the antibiotic unable to interact with its target. For example, the most common mechanism of resistance to β‐lactams is enzymatically destroy of structural β‐lactam rings that result in drug inactivation.^24^ The resistance to an antimicrobial agent can be mono (caused by a unique mechanism) or multifactorial (caused by more than one mechanism simultaneously). For example, some strain of carbapenems‐resistant *P. aeruginosa* commonly have different mechanisms, including the production of carbapenemase, decreased expression or mutation of the outer membrane porin (OprD) and overexpression of efflux‐pumps.^22^, ^25^, ^26^

The mechanisms of resistance may burden a fitness cost to organisms. However, this cost can be decreased in the absence of antimicrobial agents by strict regulation of gene expression.^27^ The expression of resistance‐related genes or point mutations in essential genes is associated with a lower growth rate in resistant strains relative to their antibiotic‐susceptible counterparts.^28^ Some strains decrease the fitness cost of antimicrobial resistance by possessing the resistance mechanisms promoted only in exposure to drugs. Bacteria with inducible antimicrobial resistance mechanisms can be more compatible compared to bacteria with resistance by constitutively expressed mechanisms or with mutations in the essential genes conferring resistance.^29^

### 3.2 Resistance evolution in the exposure to antimicrobial agents

**Figure 2:**
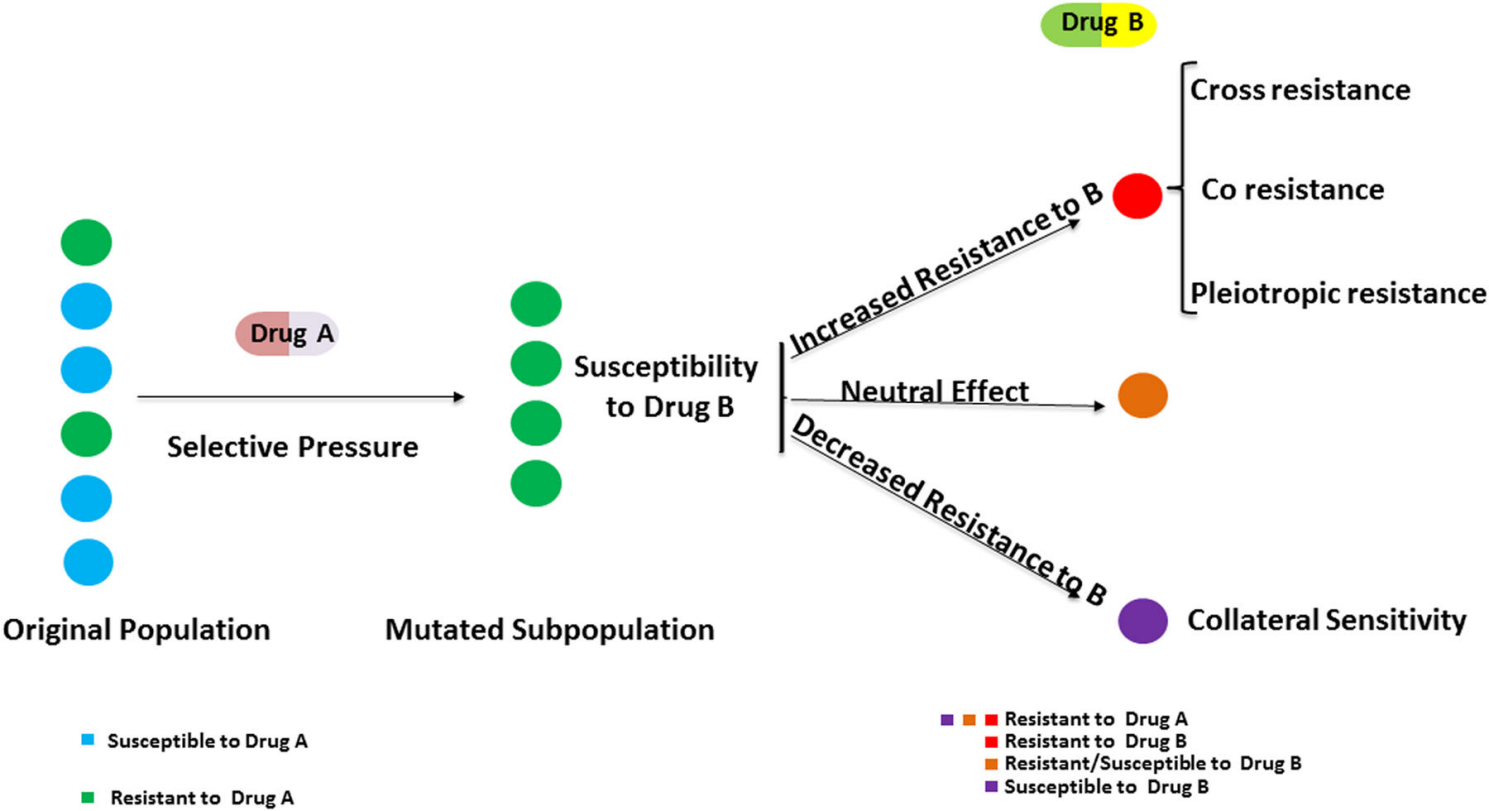
Evolutionary trade‐offs between antimicrobial agents: The ability of bacteria to adaptation to different conditions is related to evolutionary exchange under new environmental conditions. In the presence of drug A, several types of evolutionary trade‐offs may happened. Increased resistance to drug A is associated with increased resistance to drug B in some mutants. Resistant mutant to drug A may display increased sensitivity to drug B.

The relations between the levels of resistance and administration of antibiotics have been demonstrated in both health care settings and community isolated bacteria.^30^ The same association has also been shown in the agriculture, in particular livestock.^31^, ^32^ The increased frequency of outbreaks caused by resistant pathogens is an anticipatable result of this condition. In contrast, the decreasing the levels of or abolishing exposure to antibiotics, a reduction of resistance frequency may happen, although it is less evident. The evolution of antibiotic resistance is developed in response to the selective pressure of antimicrobial agents.^33^ These evolutionary theories govern the resistance to different agents in microbial pathogens and cancer cells.^34^, ^35^ In the absence of antimicrobial agents, molecular mechanisms of antimicrobial resistance frequently underpin fitness.^33^ Different mechanisms of drug resistance may affect one another to trigger non‐additive fitness interactions, an event referred to as epistasis.^36^ These interactions can stimulate uneven fitness features, potentially limiting the frequency of available evolutionary tracks to elevated adaptability or making evolution irreversible.^37^ Although resistance to antimicrobial agents may be beneficial to microorganisms, some data has been shows that drug‐resistant microorganisms suffer a high fitness cost.^38^, ^39^, ^40^ The potential of bacteria in rapid adaptation to different conditions is often dependent on evolutionary trade‐offs in the form of decreased fitness under new environmental conditions.^41^ In the exposure to antimicrobial agents, two types of evolutionary events are common (Figure 2): (a) increased resistance that is high‐cost in the absence of the antimicrobial agents, causing cellular deficiencies than the original susceptible parent, and (b) evolved resistance may increase susceptibility to other agents.^42^

#### 3.2.1 Cross‐resistance to antimicrobial agents

Cross‐resistance is defined as the resistance to all agents assigned in the same class of antibiotics through a common mechanism. Importantly, cross‐resistance implies cross‐selection; the use of a given antibiotic can select resistance to other members of the same class, but not to drugs belonging to different classes. Antibiotics belonging to the same category are chemically similar, have therefore, the same cellular targets, and show cross‐resistance.^43^ The levels of cross‐resistance are different for members of a drug class. Recently developed agents are commonly more effective than elderly members of the same group of drugs are. For example, among β‐lactams, carbapenems, due to the high level of resistance to β‐lactamases, are more effective than penicillin and cephalosporins. Gram‐negative organisms that have extended‐spectrum β‐lactamases (ESBLs) become much more susceptible to carbapenems (that have low MICs) than to penicillins (that have higher MICs).^44^ This finding indicates a resistance mechanism has no definite effect on the different agents assigned in the same class.

#### 3.2.2 Coresistance to antibiotics

Co‐resistance is defined as the resistance to two or more antibiotics from different classes by accumulating different mechanisms encoded by mutated or acquired genes.^43^ For example, MRSA, in the results of accumulating resistance mechanisms, can be co‐resistance to others, β‐lactams, macrolides, tetracycline, aminoglycosides, and fluoroquinolones.^45^, ^46^ Coresistance to non‐β‐lactams antibiotics (tetracycline, fluoroquinolones, and aminoglycosides) also has been reported among ESBLs producing Gram‐negative bacteria that are mediated by integrons and plasmids.^47^ Different antibiotics may induce coselection of isolates with coresistance. Therefore, antibiotic combination therapy might promote coresistance and coselection procedures that enhance the chance of developing persistent isolates.^43^

#### 3.2.3 Pleiotropic resistance

Pleiotropic resistance is the resistance to different classes of drugs due to the identical mechanism, such as mutation or gene acquisition.^43^ Mutations in regulatory genes cause overexpression of Mex‐AB efflux pump in *P. aeruginosa*, that confers resistance to β‐lactam agents, fluoroquinolones, and aminoglycosides.^22^

#### 3.2.4 Collateral sensitivity

In an evolutionary trade‐off known as collateral sensitivity, the resistance mechanism to a certain antibiotic(s) may be mediated by the hypersensitivity to other drugs. Collateral sensitivity implies reverse cross‐resistance to antimicrobial agents and is a typical pleiotropic result of resistance evolution due to mutation in and, or acquisition of resistance genes.^16^, ^48^ Collateral sensitivity frequently arises between two agents with different microbial inhibitory mechanisms. For example, the antibacterial activity of β‐lactam agents is commonly increased in aminoglycoside‐resistant *Escherichia coli*.^49^, ^50^

### 3.3 Collateral sensitivity: An attractive option for dealing with drug‐resistant pathogens

Collateral sensitivity frequently occurs through the evolutionary trade‐off of bacteria because of resistance to antibiotics. According to an in vitro study, 74% of laboratory‐evolved resistant strains to certain antibiotic have shown collateral sensitivity to one or more antimicrobial agents.^51^ Therefore, the idea emerges whether collateral sensitivity effect can be applied to battle the drug resistance crisis and control pathogens adapting to multiple antimicrobial agents. Resistant pathogens may be efficiently treated by altering a drug to other antimicrobial agent that the bacteria show collateral sensitivity to it. Successive multiantibiotic therapy with cycles between antimicrobial agents with collateral sensitivity may significantly decrease the emergence of resistance. A developing resistant strain is deleted by switching of agents that are effective due to collateral sensitivity. This procedure could be sequentially repeated in a serial administration of multidrug to keep drugs susceptibility in the bacteria. A possible application of such serial antibiotic administration has been described by some in vitro studies against some infectious pathogens.^52^ Collateral sensitivity has even seen between therapeutic antibiotics and others compound with microbial inhibitory effects such as antimicrobial peptides (AMPs).^48^

### 3.4 Mechanisms confer collateral sensitivity: What has been reported until now

The molecular mechanisms of collateral sensitivity have not been well understood. Given the frequency of mutations‐ dependent resistance with multiple phenotypic expressions, multidrug resistance can increase sensitivity to other groups of the antimicrobial agent as a side effect. Different resistance mechanisms, such as target modification and mutations, which influence antibiotics uptake and pumping out, can be potentially associated with collateral sensitivity. The most known example regards the effects of membrane‐potential‐modifying mutations.^49^ Cellular uptake of the aminoglycoside class is unique because it needs active proton motive force (PMF). Therefore, mutations of the genes that encode membrane potential, through the decrease of PMF, can be partly conferred resistance to aminoglycosides agents.^53^ These modifications affect oxidative phosphorylation, heme biosynthesis, or proton/potassium transport, resulting in decreased aminoglycosides uptake in resistant strains. However, resistance to aminoglycosides due to the mutations of PMF potential membrane proteins gives rise to a high cost for bacteria, because the resistance to several other antimicrobial agents is mediated by PMF‐dependent efflux pumps.^15^, ^49^ Indeed, decreaseing PMF in aminoglycoside‐resistant strains can reduce the protective effects of PMF‐dependent efflux pumps and thus lead to hypersensitivity to different classes of antimicrobial agents. The multidrug resistance AcrAB efflux pumps, an RND‐type efflux system in *E. coli*, are significantly influenced in aminoglycosides resistant mutants due to decreased PMF and are associated with collateral sensitivity interaction.^49^ Therefore, the mutation in the PMF is a double‐edged sword because effects the intra‐bacterial drug levels in an antagonistic mode. Some studies have been indicated the *tet* gene is commonly transferred by a plasmid‐mediated pathway between pathogens.^54^ The *tet* gene expression not only mediates resistance to tetracycline due to pump it out of bacteria, it synchronously induces hypersensitivity of Gram‐negative pathogens to aminoglycoside agents by increasing the cellular uptake of these drugs.^55^ The expression of *tet* genes is not associated with an effect on the membrane potential, indicating that this expression may enhance aminoglycosides uptake by affecting the availability of particular transporter or by decreasing the minimum potential of the membrane that is needed for uptake. More commonly, the effect on gene expression because of resistance to antimicrobial agents can be an inducer of collateral sensitivity. In this regard, the most common mechanism of fluoroquinolones resistance is mutations in the target topoisomerase gene (*gyrA*). Topoisomerase regulates the DNA supercoiling of bacteria.^56^, ^57^ One of these clinically important mutations (Asp87Gly) causing resistance to fluoroquinolones, unexpectedly increases the susceptibility to some other classes of drugs, such as doxycycline and nitrofurantoin.^58^ The possible effect is that mutations (Asp87Gly) alter DNA gyrase activity (DNA supercoiling), and such modification affects transcription across the genome. DNA supercoiling also influences the transcription of different genes critical in the survival of bacteria in response to stress conditions (such as *rpoS* and *recA*).

Imamovic et al.^59^ reported a complex collateral sensitivity network between various antimicrobial agents in *P. aeruginosa* strains. According to their results, bacterial populations exposed to azithromycin and ciprofloxacin have a significant tendency to *nfxB* mutation. Resistant PAO1 strains to quinolone, macrolide, and tetracyclines had mutations in the pathoadaptive gene *nfxB*.^60^, ^61^ NfxB is a negative transcriptional regulator of the MexCD‐Oprj efflux system. Overexpression of MexC transporter protein has been observed in both ciprofloxacin‐resistant and azithromycin‐resistant isolates.^62^, ^63^ *nfxB* mutations lead to an overexpression of the MexC transporter leading to collateral sensitivity toward aminoglycosides, β‐lactams, and colistin.

Lindquist et al. studied in vivo development of *Candida albicans*, a clinically important fungal pathogen, resistant to amphotericin B.^64^ The resistance of *C. albicans* to amphotericin B is uncommon, even with prolonged and frequent use of this agent. This could be described in two ways. Mutation‐mediated resistance may arise at eitherlow frequency or cause high compatibility costs in the host environment—and are thus selected against. Moreover, mutation‐mediated amphotericin B resistance induces cellular stress that triggers increased expression of the cellular chaperone Hsp90. As a result, these mutations are associated with hypersensitivity of strains to environmental stress, and host immune defense, deficiency in filamentation and pathogenesis.^64^ Therefore, trade‐offs related to resistance may arise not only between sensitivity to various antimicrobial agents but also can occur between resistance and pathogenesis.

For the gentamicin‐adapted populations, hypersensitivity to piperacillin‐tazobactam and carbenicillin has been reported to be associated with mutations in *pmrB*.^65^ PmrB is a sensor kinase involved in the resistance to cationic antibacterial peptides, polymyxins, and aminoglycosides in *Klebsiella pneumoniae*, *Salmonella* and *P. aeruginosa*.^66^, ^67^, ^68^ The *pmr*B‐depended aminoglycoside resistance is regulated by two systems (PhoP/PhoQ and PmrB/PmrA) in response to low Mg^2+^ conditions and high (100 µM) concentrations of Fe^3+^ or by reduced pH.^69^, ^70^ The induction of PhoP/‐PhoQ and PmrB/‐PmrA regulatory system finally modify lipid A in the cell wall, including the addition of N_4_‐aminoarabinose, ethanolamine, palmitic acid and that decrease of the negative charge of bacterial membrane.^71^, ^72^ Such modification, have been reported to be associated increased sensitivity to β‐lactams in aminoglycoside‐resistant bacteria.^65^, ^73^

Collateral sensitivity to nitrofurantoin has shown to be mediated by increased drug toxicity due to interference of the SOS response in bacteria.^16^ The SOS response is an inducible mechanism mainly regulated by a transcriptional repressor (LexA) and a recombinase (RecA).^14^, ^74^ The SOS response is a well‐known DNA damage response in bacteria.^14^ The suppressor of Lon (SulA) is a cell‐division inhibitor involved in the SOS response that SOS repressor (LexA) regulates its expression. Induction of *sulA* expression is part of the late SOS response, and increased SulA levels stop replication and allow the cell to perform DNA lesion repair. SulA is degraded by the Lon protease, and cell growth eventually resumes. It has been demonstrated that nitrofurantoin causes DNA damage and induces the canonical SOS response in *E*. *coli*.^75^ Collateral sensitivity to nitrofurantoin is observed in spontaneous mutants with resistance to tigecycline (*lon* mutation).^16^ Induction of the SOS response by nitrofurantoin may contribute to the collateral sensitivity of the *lon* mutant. Upon induction of SOS, SulA would accumulate and not be degraded in a *lon* mutant, thereby blocking cell division and increaseing antibiotic toxicity due to interference of a native drug‐response system with growth.^16^

Colistin (polymyxin E) is a cationic lipopeptide with antibacterial activity against Gram‐negative bacteria.^76^ It interacted with the lipid A moiety of lipopolysaccharide (LPS) and disturbes the outer membrane of the target bacteria. Two important mutations that caused colistin resistance in *Acinetobacter baumannii* are mutations in the pmrAB locus that cause modification in ethanolamine modification of lipid A and increase the positive charge of LPS; and the mutations of the *lpxA*, *lpxC* and *lpxD* genes that cause loss of LPS formation.^77^ Given the vital role of the outer membrane as a barrier to the entry of extracellular molecules, colistin resistance through the mutation of *lpxA, lpxC* and *lpxD* genes in *A. baumannii* can influence the susceptibility to other antimicrobial agents. Dramatically increased susceptibility to azithromycin, rifampicin, and vancomycin and moderately increased resistance to amikacin, ceftazidime, imipenem, cefepime and meropenem have been demonstrated in the LPS‐deficient mutants than parental strains.^78^

LpxC is a zinc‐dependent deacetylase involved in the primary step of lipid A biosynthesis. LpxC inhibitors have been suggested for design as an antimicrobial agent.^79^ PF‐5081090 is a pyridone methylsulfone hydroxamate‐based LpxC inhibitor commercially available.^80^ Interestingly, decreased levels of LPS in the presence of PF‐5081090 increase the resistance to colistin and increase susceptibility to rifampin, vancomycin, azithromycin, imipenem, and amikacin.^81^

Recently a convergent phenotypic evolution has been observed towards collateral sensitivity to fosfomycin for resistant mutants of *P. aeruginosa*, developed in the exposure to a different class of antimicrobial drugs, including tobramycin, tigecycline, or ceftazidime. The mechanisms of the collateral sensitivity to fosfomycin have reported a decreased expression of genes encoding the peptidoglycan‐recycling pathway, which conserves the peptidoglycan formation in conditions where its de novo formation is inhibited, and a decreased expression of the *fosA* gene, which encodes a fosfomycin‐inactivating enzyme.^82^

### 3.5 Collateral sensitivity ‐informed treatment; developing, advantages, and limitations

Collateral sensitivity frequently occurs through the evolutionary trade‐off of bacteria because of resistance to antibiotics. According to an in vitro study, 74% of laboratory‐evolved resistant strains to certain antibiotic have shown collateral sensitivity to one or more antimicrobial agents.^51^ Therefore, the idea emerges whether collateral sensitivity effect can be applied to battle the drug resistance crisis and control pathogens adapting to multiple antimicrobial agents. Resistant pathogens may be efficiently treated by altering a drug to other antimicrobial agents that the bacteria show collateral sensitivity to they. Successive multiantibiotic therapy with cycles between antimicrobial agents with collateral sensitivity may significantly decrease the emergence of resistance. A developing resistant strain is deleted by switching agents with collateral sensitivity. This procedure could be sequentially repeated in a serial administration of multidrug to keep drug susceptibility in the bacteria. A possible application of such serial antibiotic administration has been described by some in vitro studies against some infectious pathogens.^52^ Collateral sensitivity has been even seen between therapeutic antibiotics and other compounds with microbial inhibitory effects such as AMPs.^48^

Several studies have been investigated the probability of determining collateral sensitivity for guiding therapeutic procedures that could decrease the frequency of antimicrobial resistant mutants, such as cycling of antibiotics or combinatory therapy. The outcome and practicality of collateral sensitivity‐based treatments are significantly depended on the conservation of collateral sensitivity among various genetic contexts. Combination therapy is a golden option for the treatment of some infectious diseases such as HIV,^83^ malaria,^84^ tuberculosis^85^ and infections caused by MDR bacteria.^86^, ^87^, ^88^

Both pharmacological effects between drugs (e.g., synergism) and evolutionary procedure of microorganisms (e.g., emerge of cross‐resistance) are believed to influence the long‐term output of the combination therapy.^15^, ^89^ Several studies on combination of antimicrobial agents against bacterial pathogens have been supported this theory. Simultaneously using of certain agents against bacteria showed a strong synergy and significantly decreased resistance evolution in *vitro* evolutionary conditions.^90^, ^91^

How synergy and collateral sensitivity influence the emergence of resistant strains in the exposure of antimicrobial combination were shown in an in vitro evolution system of *E. coli* adapting to several antimicrobial agents and their combinations.^92^ The character of antibiotic interactions in a combination (i.e., synergistic, additive, or antagonistic) did not have affect the frequency of resistance evolution during exposure to antimicrobial agents, not least due to the interactions themselves being affected by the evolutionary procedure. In contrast, the incidence of collateral sensitivity between two agents has been reported as an effective predictor of reduced development resistance through concurrent treatment by two or more antibiotics.^92^ These laboratory findings show that synergy interaction can facilitate to give an appropriate control of bacteria in the primary step of combination treatment.

Drug cycling is a strategy proposed to decrease the frequency of antimicrobial resistance that is defined as the programmed rotation of one antimicrobial agent with other different classes that display similar activity spectra.^93^

Collateral sensitivity has been identified between various antimicrobial agent pairs, and it has been described that cyclical usage of one such pair decreases the in vitro evolution of resistance to either drug. There has been observed that the sensitivity levels of the wild type were not changed by periodic elimination of resistant strains through treating the collateral‐sensitive pair.^51^ Collateral sensitivity for a short switching time (i.e., one reported cycled exposure to antimicrobial day) has been shown significantly decreased the evolution rate of resistant strains.^65^, ^94^

DNA sequencing of in vitro evolved strains has been shown that, during drug cycling, mutations occurred in more genes than mutated genes in strains that are exposed to corresponding single‐antibiotics.^95^

The experimental scheme of evolutionary trade‐offs between antimicrobial agents could serve unique data in the drug combinations. However, the utility of collateral sensitivity for clinical usage is limited by several factors. Predictability is an important factor determining the potential of clinical application of collateral sensitivity for a rational design of treatment strategies.^50^ Significant differences have been reported among populations of bacteria adapted to the same antimicrobial agents. For example, all three modes of trade‐off, cross‐resistance, collateral sensitivity, and neutral effects, to gentamicin have been observed in cefsulodin‐adapted populations of *P. aeruginosa*.^65^ The dissimilarity of collateral sensitivity patterns can be because of the differences in the molecular aspect and mechanisms of antimicrobial agents and resistance. What makes the collateral sensitivity patterns difficult to predict is the prior evolution and adaptations that may influence developing resistance, cross‐resistance, and collateral sensitivity. In principle, the development of collateral sensitivity should be repeatable in a certain condition. Several studies even have been reported contrasting evolutionary trade‐offs (i.e., some evolved strains show collateral sensitivity and some show cross‐resistance or neutral effects) for different organisms, including *P. aeruginosa*,^65^ *E. coli* ^50^, ^96^ and *Enterococcus faecalis*.^97^ These findings indicate the various resistance mechanisms may result in contrasting patterns of collateral effects.^98^ Therefore, a systematic study of various pathogen/agent pairs is helpful to a fully understood incidences, and discrepancies of evolved collateral sensitivity. There was a considerable difference in the trade‐off of different species following antibiotic resistance evolution. For example, ciprofloxacin‐resistant *P. aeruginosa* strains have been exhibited different collateral sensitivity patterns,^65^ whereas ciprofloxacin resistance has been shown to show hypersensitivity to the same drugs in *E. coli*.^49^, ^51^ These different patterns can be the result of the stochastic character of mutations as well as multiple evolutionary paths to resistance to the first‐line antimicrobial agent, subsequently impact on the susceptibility to other agents. The evolutionary trade‐offs are not always repeatable across conditions. For example, a drug pair, which repeatedly induces collateral sensitivity in the small populations of certain bacteria, may cause a cross‐resistance effect in larger populations of same bacteria. Therefore, bacteria could evade the evolutionary pressure, likely because of a higher possibility of mutations with a favorable outcome for the survival of bacteria under these conditions. The ability of bacteria to evolve to overcome collateral sensitivity is an important concern for applying collateral sensitivity, which is not well understood. This implies that the evolutionary trade‐off should be stable across time and microorganisms cannot evolve to overcome collateral sensitivity. If bacteria acquire resistance to antimicrobial agent B, they should show collateral sensitivity to the primary drug A (uni‐directional collateral sensitivity).^65^

## 4 CONCLUSION

Collateral sensitivity can be a guide to an effective strategy in dealing with antibiotic resistance if selected agents are cycled or combined optimally. Its application as a guide for treatment is based on the theory that the exploited evolutionary trade‐off is permanent and predictable. Several studies have anticipated collateral sensitivity‐based cycling therapy in the clinic can restore control of infections caused by MDR bacteria. Despite some progress in the findings of collateral sensitivity, there are more unknown key points for addressing in the future. Primarily the fitness cost of HGT and plasmid‐mediated resistance to antibiotics is of paramount significance. The repeatability of collateral sensitivity in a particular condition and across different conditions needs studied more. The ability of bacteria in evolve to overcome collateral sensitivity is an essential factor for the application of collateral sensitivity, which is not well understood. This stochasticity in the collateral sensitivity networks precludes a general use of this evolutionary trade‐off, since it can be exploited just when robust collateral sensitivity patterns are found.

## AUTHOR CONTRIBUTIONS

**Mina Yekani**: Conceptualization; investigation; methodology; software. **Robab Azargun**: Investigation; methodology; writing—original draft. **Simin Sharifi**: Data curation; methodology; software; writing—original draft. **Edris Nabizadeh**: Investigation; methodology; writing—original draft. **Javid Sadri Nahand**: Investigation; methodology; writing—original draft. **Navideh Karimi Ansari**: Investigation; methodology; software; writing—original draft. **Mohammad Yousef Memar**: Conceptualization; investigation; validation; writing—original draft; writing—review and editing. **Jozsef' Soki**: Conceptualization; investigation; methodology; supervision; writing—original draft; writing—review and editing.

## CONFLICT OF INTEREST STATEMENT

The authors declare no conflict of interest.

## ETHICS STATEMENT

This article does not contain any studies with animals performed by any of the authors.

## TRANSPARENCY STATEMENT

The lead author Mohammad Yousef Memar, Jozsef' Soki affirms that this manuscript is an honest, accurate, and transparent account of the study being reported; that no important aspects of the study have been omitted; and that any discrepancies from the study as planned (and, if relevant, registered) have been explained.

## Supporting information

Supporting information.

## Data Availability

Data sharing is not applicable to this article as no new data were created or analyzed in this study.
