# Supplementary material for: Collateral sensitivity: An evolutionary trade‐off between antibiotic resistance mechanisms, attractive for dealing with drug‐resistance crisis
Source: Health Sci Rep. 2023 Jul 11;6(7):e1418. doi: 10.1002/hsr2.1418 (PMC10336338; doi:10.1002/hsr2.1418)
Supplement: Supplementary file 1 — Supporting information. [file HSR2-6-e1418-s001.docx]

Flow chart of the literature selection process in the present articles

-Excluded studies

-Review and congress abstract

-Studies not reporting

Excluded Studies

Title and abstract screening

Studies detected by the initial screening of databases: n=

Google scholar

PubMed

Included studies

Full text

Duplication

Scopus
